# Annotations

**Published:** 1891-06-20

**Authors:** 


### Annotations.

**The Poor Clergyman's Holiday.**

The clergy who work in the poorer districts of London and other large towns have a toilsome and dreary life. They are men who, for the most part, have received a University education, and they often live on the wages of third-rate clerks and skilled operatives. That they perform their work with diligence, and possess their souls in patience under such circumstances speaks volumes for their religious faith and for their sturdy manhood. It is a stern task to live like a gentleman and bring up a family in London on less than two hundred a-year. This is the task to which many of the clergy have to address themselves. There are three things that are essential for the working Londoner—food, a home, and country holidays. No man can keep, even approximately well, who does not get out of London for at least three or four weeks every year. Still less can any man be efficient as a fresh thinker, a teacher of religion, and a moral guide and example to the people unless he has that indispensable requisite, sound physical health. Such health is impossible without a periodical deliverance from the noise, the bad air, the strenuous toil, and the wearing anxiety of an ill-paid London life. It is a melancholy thing to think of that a public and charitable fund is necessary in order that some of the harder worked clergy and their families may secure their indispensable summer holiday. But, unfortunately, there is no other way of deliverance, and it is better that the clergy should have a holiday by the help of the benevolent than that they should not have a holiday at all. Prebendary Ingram makes an appeal in the morning papers on behalf of the Clergy Holiday Fund. The Fund is administered privately by the Archdeacons of London and Middlesex, and Prebendary Ingram undertakes the collection of the money. Not one of those three gentlemen charges anything for his services. There is not even an office and a clerk to be paid for out of the collected money. The Prebendary's own house, St. Margaret's Rectory, 20, Finsbury Square, is the place to which subscriptions may be sent. For ourselves we thank the Reverend Prebendary Ingram and the Archdeacons of London and Middlesex for the sternly necessary and truly admirable work they have undertaken on behalf of their poorer brethren. Those who have money to spare cannot possibly make a better use of it than by sending a subscription to the Clergy Holiday Fund. Religion, and a religious example, are as necessary for the poor as clothing or daily bread. Without a body of separated clergymen religion on a large scale would seem to be impossible. To the poorer clergy the country owes a debt which it will never repay. Let us try to repay at least a little part of it by giving such faithful public servants a brief summer holiday.

**Why Men Gamble.**

Science loves to ask questions. A true science asks them everywhere and at all times, and of every kind of person who is the least likely to have an answer to give. The Bishop of Durham, Dr. Westcott, says he knows why men gamble. It is not unlikely that the Bishop really does know something about the subject, and we shall be glad to interrogate him. He has many qualifications for such knowledge. He is familiar with the life of English public schools and English Universities. He is, moreover, a man of large sympathies and of honest and inquiring mind; he is both learned and scientific in a high degree. In short, if one were called upon to select an ideal person to give a dispassionate answer to the question why men gamble, one would hardly know where to look for a fitter man than Dr. Westcott. The Bishop says that most men gamble because their lives are so "aimless." They have no business to conduct, no important purposes on hand, and so they become mentally dull and weary. In this state of mind gambling offers them a little easy excitement; hence they gamble. Now, it need not be assumed that gamblers are more wicked than other people; as a rule they probably are not; they are only more idle, more feeble-minded, more inconsequent. The Bishop of Durham has hit upon an important physiological truth. The human brain differs essentially from that of the dog, of the monkey, of the horse, or any of the higher animals. These creatures can act with intelligence on the spur of the moment, and in obedience to a sufficiently strong stimulus; but the man of the higher races can only be said to live at all as a man when he is the maker and executant of definite purposes. No really able man now-a-days thinks that idleness is the proper calling of a gentleman. The worthier members of the aristocracy all admire work and seek careers for themselves as eagerly as do the members of other classes. The idea that idleness is a mark of distinction may still linger among the sillier and less educated portion of the middle and trading classes. But even the new rich are beginning to have the sense to be ashamed of merely eating and drinking, and dressing themselves to be looked at. Physiology joins hands with morals in the effort to make men think like men and act like men. If the moralist is justly angry with the idler and the gambler, the physiologist is scientifically impatient and contemptuous. He cannot see without anger one of the worthiest pieces of divine mechanism lying, so to speak, on the roadside of idleness, and rotting like the broken-off branch of a noble tree. To him the idler and the gambler is a rotting and a worthless branch. There is no doubt that more and more physiology will take the side of morals, and will denounce with more than religious fervour the stupid, the idle, and the vicious who deface and destroy in their own persons some of the greatest of all Creation's works.

**Laundresses on Protest.**

Good-natured England has long taken a lively interest in the efforts of women to secure for themselves a manly, or rather womanly, independence. Such efforts are often accompanied by unfamiliar and sometimes decidedly unconventional demonstrations. The laundresses in Hyde Park, on Sunday, behaved themselves in a very un-Belgravian fashion. It is very likely that a gathering of negro women in the centre of Africa would have conducted themselves with more gravity and sobriety than did some of the cockney lasses who came out to ventilate their grievances on the green sward. But we must not permit our somewhat insular passion for severe external propriety to hide from us the real and serious grievances of which London laundresses have to complain. They work very hard, and their hours are extremely long. The places in which their trade is carried on are often so small and stifling as to be absolutely poisonous; and their pay, considering the length of their hours and the sternness of their labours, is exceedingly meagre. We who pay the proprietors of laundries know very well that we are charged smartly enough, whoever pockets the money. We do not grudge what we pay, or at least we should not grudge it if we knew that a just proportion went into the pockets of the poor creatures who slave at the wash-tub or wear out their strength and life at the ironing-table. The women demand the protection of the Factories Acts—a most reasonable demand; and we are glad to see that of the large number of Members of Parliament who have been appealed to, almost nine in every ten have promised their countenance and help. Speaking generally, there is no doubt that the more labour is organised and the more clearly its claims can be stated before public opinion the better is it, not only for labour itself, but also for the employers of labour, and for the whole community. Somebody long ago said that "Nature abhors a vacuum." If he had said that "Nature abhors a chaos," he would have been very much nearer the truth. Organisation and specialisation on right lines are the prime conditions of successful life and government for large populations. We congratulate the laundry women on their first, but most successful effort at forming themselves into an ordered and effective community for self-help.

